# Shaping the research agenda of pediatric gastroenterology, hepatology, and nutrition: The proposal from an Italian Society of Pediatric Gastroenterology, Hepatology and Nutrition (SIGENP) expert group

**DOI:** 10.1002/jpr3.70228

**Published:** 2026-08-25

**Authors:** Carlo Agostoni, Daniele Alberti, Renata Auricchio, Maria Elisabetta Baldassarre, Osvaldo Borrelli, Matteo Bramuzzo, Teresa Capriati, Carlo Catassi, Sabrina Cenni, Marco Crocco, Salvatore Cucchiara, Luigi Dall'Oglio, Paola De Angelis, Marco Deganello Saccomani, Fabiola Di Dato, Angelo Di Giorgio, Antonella Diamanti, Valeria Dipasquale, Enrico Felici, Ruggiero Francavilla, Valentina Giorgio, Luigi Greco, Raffaele lorio, Flavio Labriola, Paolo Lionetti, Elena Lionetti, Giuseppe Maggiore, Monica Malamisura, Claudia Mandato, Antonio Marseglia, Emanuele Nicastro, Lorenzo Norsa, Salvatore Oliva, Licia Pensabene, Elena Sofia Pieri, Paolo Quitadamo, Giusy Ranucci, Sara Renzo, Claudio Romano, Silvia Salvatore, Luca Scarallo, Maria Immacolata Spagnuolo, Annamaria Staiano, Caterina Strisciuglio, Renato Tambucci, Riccardo Troncone, Pietro Vajro

**Affiliations:** ^1^ Fondazione De Marchi ‐ Pediatric Area, IRCCS Ca’ Granda Ospedale Maggiore Policlinico Milan Italy; ^2^ Department of Clinical Sciences and Community Health University of Milan Milan Italy; ^3^ Department of Pediatric Surgery, ASST Spedali Civili Children Hospital University of Brescia Brescia Italy; ^4^ Department of Clinical and Experimental Sciences University of Brescia Brescia Italy; ^5^ Department of Translational Medicine & European Laboratory for the Investigation of Food‐Induced Diseases University Federico II Naples Italy; ^6^ Department of Interdisciplinary Medicine‐ Neonatology and NICU Section University “Aldo Moro” of Bari Bari Italy; ^7^ Department of Paediatric Gastroenterology Great Ormond Street Hospital for Children London UK; ^8^ Institute for Maternal and Child Health ‐ IRCCS “Burlo Garofolo” Trieste Italy; ^9^ Digestive Diseases and Rehabilitation Clinical Unit; Nutritional Treatments of Complex Disease Research, Unit of Pediatric, Hospital Bambino Gesù Rome Italy; ^10^ Department of Pediatrics Polytechnic University of Marche Ancona Italy; ^11^ Pediatric Emergency Unit, Santobono‐Pausilipon Children's Hospital, AORN Naples Italy; ^12^ Pediatric Gastroenterology and Endoscopy Unit, IRCCS Istituto Giannina Gaslini Genova Italy; ^13^ Sapienza Università di Roma Roma Italy; ^14^ Bambino Gesù Children's Hospital Rome Italy; ^15^ Digestive Endoscopy and Surgery Unit Bambino Gesù Children's Hospital Rome Italy; ^16^ Department of Paediatrics Woman's & Child's University Hospital of Verona Verona Italy; ^17^ Dipartimento di Scienze Mediche Traslazionali Sezione di Pediatria Università degli Studi di Napoli Federico II Naples Italy; ^18^ Department of Medicine University of Udine Udine Italy; ^19^ Digestive Diseases and Rehabilitation Clinical Unit; Nutritional Treatments of Complex Disease Research Unit of Pediatric Hospital Bambino Gesù Rome Italy; ^20^ Department of Human Pathology in Adulthood and Childhood “G. Barresi”, Pediatric Gastroenterology and Cystic Fibrosis Unit University of Messina Messina Italy; ^21^ Pediatric and Pediatric Emergency Unit, Children's Hospital, AOU SS Antonio E Biagio E C. Arrigo Alessandria Italy; ^22^ Pediatric Gastroenterology and Hepatology Unit, Department of Interdisciplinary Medicine, Children's Hospital‐Giovanni XXIII University of Bari Aldo Moro Bari Italy; ^23^ Department of Women, Children and Public Health Sciences, Fondazione Policlinico Universitario “A. Gemelli” IRCCS Università Cattolica del Sacro Cuore Rome Italy; ^24^ European Laboratory for Food‐Induced Disease University of Naples Federico II Naples Italy; ^25^ Dipartimento di Scienze Mediche Traslazionali Epatologia Pediatrica Università di Napoli Federico II Naples Italy; ^26^ Pediatric Gastroenterology Unit, Maggiore Hospital Bologna Italy; ^27^ Department Neurofarba University of Florence, Meyer Children's Hospital IRCCS Florence Italy; ^28^ Department of Pediatrics Marche Polytechnic University Ancona Italy; ^29^ Division of Hepatogastroenterology and Liver Transplant, ERN RARE LIVER, Bambino Gesù Children's Hospital, IRCCS Rome Italy; ^30^ Gastroenterology and Nutrition Unit, Bambino Gesù Children's Hospital, IRCCS Rome Italy; ^31^ Dipartimento di Medicina, Chirurgia e Odontoiatria “Scuola Medica Salernitana”, Section of Pediatrics Baronissi (Salerno) Italy; ^32^ Fondazione IRCCS Casa Sollievo della Sofferenza San Giovanni Rotondo (Fg) Italy; ^33^ Hepatology, Gastroenterology and Transplantation Unit, Hospital Papa Giovanni XXIII Bergamo Italy; ^34^ Department of Pediatrics, Vittore Buzzi Children's Hospital University of Milan Milan Italy; ^35^ Pediatric Gastroenterology and Liver Unit, Maternal and Child Health Department Sapienza ‐ University of Rome Rome Italy; ^36^ Pediatric Unit, Department of Medical and Surgical Sciences University “Magna Graecia” of Catanzaro Catanzaro Italy; ^37^ Pediatric Unit, San Donato Hospital Arezzo Italy; ^38^ Department of Pediatrics Santobono‐Pausilipon Children's Hospital Naples Italy; ^39^ Department of Pediatrics, Liver Transplant Center UPMC‐ISMETT Palermo Palermo Italy; ^40^ Gastroenterology and Nutrition Unit, Meyer Children's Hospital Florence Italy; ^41^ Department of Human Pathology in Adulthood and Childhood “G. Barresi” Pediatric, Gastroenterology and Cystic Fibrosis Unit University of Messina Messina Italy; ^42^ Pediatric Unit, Hospital “F. Del Ponte”, Department of Medicine and Technical Innovation University of Insubria Varese Italy; ^43^ Gastroenterology and Nutrition Unit, Meyer Children's Hospital IRCCS Florence Italy; ^44^ Department of Translational Medical Sciences University “Federico II” of Naples Naples Italy; ^45^ Department of Translational Medical Sciences‐Section of Pediatric University Federico II Naples Italy; ^46^ Department of Woman, Child and General and Specialistic Surgery University of Campania “Luigi Vanvitelli” Napoli Italy; ^47^ Gastroenterology and Nutrition Unit, Bambino Gesù Children's Hospital IRCCS Rome Italy; ^48^ Department of Medical Translational Sciences and European Laboratory for the Investigation of Food‐Induced Diseases University Federico II Naples Italy; ^49^ Department of Medicine, Surgery and Dentistry “Scuola Medica Salernitana” University of Salerno Salerno Italy

**Keywords:** multicenter collaboration, pediatric gastroenterology research, precision medicine, real‐world evidence, translational science

## Abstract

The Italian Society of Pediatric Gastroenterology, Hepatology and Nutrition (SIGENP) recognized the need to define research priorities and identify knowledge gaps to guide future investigations in pediatric digestive health. Following international examples, SIGENP aimed to provide a structured framework that aligns scientific innovation with clinical relevance and patient‐centered outcomes. This narrative review represents the consensus output of SIGENP's thematic working groups (WGs) in key fields—inflammatory bowel disease (IBD), Gastrointestinal Endoscopy, Hepatology, Nutrition and Intestinal Failure, Functional and Motility Disorders, and Allergy and Immunity. Each WG conducted a focused review of recent literature, national activities, and registry data, identifying unmet needs and defining strategic objectives. Draft reports were refined through plenary discussions and finalized by consensus during dedicated SIGENP meetings. Across all areas, shared priorities emerged: advancing precision and personalized medicine, integrating multi‐omics and digital tools, and promoting pediatric‐specific evidence through multicenter collaboration and harmonized registries. Disease‐specific gaps include early diagnosis and biomarker discovery in IBD and liver disease, standardization of pediatric endoscopy and motility diagnostics, optimization of nutritional interventions and intestinal failure management, and the exploration of organoid and immune models in celiac disease and food allergy. This review outlines a comprehensive research agenda in pediatric gastroenterology, hepatology, and nutrition developed through a structured SIGENP expert process. It provides a strategic framework to guide future research initiatives, foster collaboration, and ultimately improve outcomes for children with gastrointestinal and nutritional disorders.

## Abstract (highlights)

**What Is Known:**

- Pediatric gastroenterology faces rising disease incidence, uneven access to care, and limited pediatric‐specific evidence.
- National and international societies increasingly emphasize research prioritization to address gaps across inflammatory bowel disease, hepatology, endoscopy, nutrition, allergy, and motility.
- Multicenter registries and translational approaches are essential to advance precision medicine in children.

**What Is New:**

- The Italian Society of Pediatric Gastroenterology, Hepatology, and Nutrition (SIGENP) provides the first integrated, cross‐disciplinary national research agenda in pediatric gastroenterology.
- The document identifies high‐impact priorities and strategic plans across six domains, emphasizing multi‐omics, non‐invasive diagnostics, artificial intelligence, and patient‐centered outcomes.
- This roadmap supports equitable, evidence‐based, and future‐ready pediatric care.

## 1 INTRODUCTION

The Italian Society of Pediatric Gastroenterology, Hepatology, and Nutrition (SIGENP) conducted an expert consensus process to identify key knowledge gaps and research priorities in pediatric gastroenterology, hepatology, and nutrition. Like other international societies, SIGENP aims to advance understanding of disease mechanisms, improve diagnostics, support non‐invasive and personalized approaches, and enhance national and international collaboration.

The research agenda focuses on six main domains—inflammatory bowel disease (IBD), motility disorders, hepatology, allergy and immunity, nutrition, and digestive endoscopy. In recent years, several collaborative networks have undertaken structured exercises to identify research priorities and knowledge gaps in pediatric gastroenterology and related fields. These initiatives aim to align clinical needs with research efforts, promote multicenter collaboration, and guide funding strategies. Within this evolving landscape, national scientific societies can contribute by translating global research challenges into coordinated initiatives while maintaining alignment with international priorities.

## 2 METHODS

This review was developed through a SIGENP‐led consensus process. Each Working Group, composed of clinicians and researchers with expertise in the respective field, conducted structured reviews of literature and ongoing research activities to identify knowledge gaps and research priorities. Preliminary priority lists were developed independently and subsequently discussed during dedicated consensus meetings. Through iterative discussions, priorities were refined according to clinical relevance, feasibility, and potential impact on pediatric care. The final research priorities and strategic directions were harmonized across Working Groups and are summarized in Table 1. The final document, therefore, represents the consensus outcome of this expert‐driven process.

**Table 1:** Research priorities and strategic plans across SIGENP working groups.

Working group	Research priorities	Strategic plans
IBD	• Identify molecular, microbial, and environmental biomarkers predictive of disease activity and treatment response. • Evaluate long‐term safety and real‐world effectiveness of biologics, small molecules, and dietary therapies across developmental stages. • Define therapeutic targets (mucosal, histologic, transmural healing) relevant to pediatric outcomes.	• Develop a harmonized nationwide network of pediatric IBD centers integrating registries, biobanks, and standardized data collection. • Promote comparative studies of pharmacologic and nutritional strategies. • Strengthen regulatory and industry partnerships to accelerate pediatric approvals and optimize PK/PD‐driven dosing.
Gastrointestinal endoscopy	• Define pediatric benchmarks for procedural safety, reporting quality, and long‐term outcomes. • Validate AI‐supported image analysis and computer‐aided diagnostic tools. • Expand access to advanced therapeutic procedures (ERCP, EUS, POEM).	• Expand the EndoPED national registry and implement AI‐assisted auditing for quality improvement. • Integrate simulation‐based curricula and competency frameworks across centers. • Strengthen collaborations with adult and international societies to harmonize training and promote equitable access.
Hepatology	• Advance gene‐ and cell‐based therapies for inherited liver disorders; define predictors of response and long‐term safety. • Standardize non‐invasive biomarkers and imaging tools for cholestatic, metabolic, autoimmune, and vascular liver diseases. • Characterize immune–microbiome interactions and determinants of chronicity.	• Establish multicenter consortia for genetic and metabolic hepatopathies. • Implement omics‐integrated prognostic models in clinical pathways. • Embed psychosocial assessment, patient‐reported outcomes, and structured transition programs in routine care.
Nutrition and intestinal failure	• Characterize early‐life determinants of metabolic and gastrointestinal health. • Evaluate comparative effectiveness of polymeric versus real‐food enteral formulas. • Optimize indications, timing, and monitoring of teduglutide and other adaptation‐enhancing therapies.	• Develop multicenter prospective cohorts and REDCap‐based registries. • Secure national and EU funding for translational nutrition projects. • Integrate early‐life nutrition research with obesity‐prevention initiatives and intestinal failure networks.
Functional and motility disorders	• Generate age‐specific reference values for advanced motility diagnostics (HRM, FLIP, colonic manometry). • Evaluate emerging therapies (neuromodulation, CBT, PENFS) through rigorous pediatric trials. • Apply AI and machine learning for motility pattern interpretation and phenotypic clustering.	• Establish multicenter registries for rare motility disorders and standardized diagnostic workflows. • Validate device‐based and digital health innovations in real‐world pediatric settings. • Strengthen neurogastroenterology training and multidisciplinary research platforms.
Allergy and immunity	• Develop patient‐derived organoid models to dissect immune–epithelial interactions in celiac disease and food‐related disorders. • Investigate early‐life environmental exposures and gene–environment interactions in immune tolerance. • Identify biomarkers for food intolerance and non‐IgE‐mediated gastrointestinal reactions.	• Launch multicenter organoid‐based translational programs integrating immunology and epithelial biology. • Establish longitudinal birth cohorts for risk stratification and prevention trials. • Promote national collaborations linking basic, clinical, and preventive allergy research.

Abbrevations: AI, artificial intelligence; CBT, cognitive behavioral therapy; ERCP, endoscopic retrograde cholangiopancreatography; EUS, endoscopic ultrasound; FLIP, functional lumen imaging probe; HRM, high‐resolution manometry; IBD, inflammatory bowel disease; PENFS, percutaneous electrical nerve field stimulation; PK/PD, pharmacokinetics/pharmacodynamics; POEM, per‐oral endoscopic myotomy; SIGENP, Italian Society of Pediatric Gastroenterology, Hepatology and Nutrition.

## 3 IBD

Pediatric IBD (pIBD), encompassing Crohn's disease (CD) and ulcerative colitis (UC), has been investigated through national and international registries that support clinical, epidemiological, and therapeutic studies. In Italy, the SIGENP IBD WG has contributed to these efforts.

Pediatric IBD results from dysregulated immune responses influenced by genetics, environment, microbiota, and host immunity. Incidence is rising,^1^ and pediatric disease is typically more extensive, aggressive, and resistant to therapy, with notable psychosocial impact.^2^ Very early onset IBD (VEOIBD) often has a monogenic basis.^3^ Management prioritizes induction and maintenance of remission while protecting growth and development.

### 3.1 Current knowledge and gaps

The Selecting Therapeutic Targets in IBD initiative established hierarchical therapeutic targets in IBD, including mucosal healing and growth optimization.^4^ Nutritional therapy remains central: exclusive enteral nutrition (EN) is the standard induction therapy, while whole‐food exclusion diets such as CD exclusion diet and the Tasty and Healthy Diet show improved efficacy and adherence.^5^ Biologic therapy has evolved through therapeutic drug monitoring (TDM), revealing that pediatric patients often require higher per‐kg dosing than adult standards, especially in VEOIBD.^6^, ^7^ Newer biologics and small molecules broaden therapeutic possibilities, though pediatric evidence remains limited and most indications are off‐label. Surgical strategies have also advanced, with early ileocecal resection appropriate in selected patients^8^ and mesentery‐including resections or Kono‐S anastomosis reducing postoperative recurrence.^9^

Key gaps include limited understanding of preclinical disease, lack of personalized dietary strategies, and insufficient TDM data and timely pediatric approval for new therapies.

Internationally, research is increasingly focused on precision medicine integrating genomics, microbiome, and immune profiling, supported by multicenter registries enabling longitudinal and patient‐centered outcomes.^2^, ^4^

### 3.2 Goals and objectives

The IBD WG aims to elucidate biological drivers of inflammation, improve early diagnosis, personalize therapy, prevent progression, and enhance quality of life. Research should clarify early disease mechanisms, including genetic and epigenetic predisposition, with polygenic models aiding risk stratification.^10^ Studies on microbiota–host interactions, epithelial barrier dysfunction, and immune pathways—including fibrosis and mesenteric involvement—are essential.^11^ Multi‐omic approaches using patient‐derived samples should guide targeted treatments. Pediatric trials should include pharmacokinetic and safety endpoints and minimize invasiveness.^12^

### 3.3 Research priorities

Priority areas include quality of life, psychosocial burden, and fatigue management. Biomarkers capable of predicting relapse, therapeutic response, and safe tapering are urgently needed. Long‐term safety of pharmacologic and dietary therapies during growth remains insufficiently understood.^13^

### 3.4 Plans

Key strategies include developing collaborative networks of pIBD centers to support clinical trials, registries, and biobanking. Comparative studies should assess pharmacologic and dietary approaches, while molecular and clinical markers must support precision medicine. Improved regulatory dialogue and pediatric‐specific trials are needed to accelerate access to new therapies. Patient and family involvement will maintain research alignment with real‐life needs.

## 4 GASTROINTESTINAL (GI) ENDOSCOPY

Over the last decade, pediatric GI endoscopy has evolved, driven by technological innovation, international guidelines, and structured training programs. National scientific societies, including SIGENP, have contributed to these developments through guidelines and training initiatives. Despite this progress, persistent challenges include the need for child‐specific equipment, standardized training, and expanded global access. The future of pediatric endoscopy depends on technological innovation, equitable resource distribution, and child‐focused strategies to optimize safety and outcomes.

### 4.1 Current knowledge and gaps

Pediatric endoscopy has expanded beyond diagnosis to include therapeutic interventions, essential for assessing and monitoring chronic IBD,^14^ eosinophilic esophagitis,^15^ and other eosinophilic gastroenteropathies,^16^ enabling personalized treatment.^17^ European and national guidelines, including SIGENP's, provide recommendations on procedures and management of foreign body ingestion and caustic injuries.^17^, ^18^

Technological advances adapted from adult practice—high‐definition and magnification endoscopy, including narrow band imaging, capsule endoscopy, miniaturized fiber‐optic systems, and nanotechnology—improve visualization, enable small bowel and biliary access, and facilitate pediatric endoscopic retrograde cholangiopancreatography (ERCP).^19^, ^20^ Minimally invasive approaches, including transnasal and trans‐PEG routes, endoscopic ultrasound (EUS),^21^ and advanced procedures such as per‐oral endoscopic myotomy (POEM), per‐oral endoscopic tumor resection, and gastric POEM (G‐POEM),^22^ expand therapeutic options. AI enhances diagnostic accuracy, automates image analysis, reduces procedure time, and supports federated learning.^23^ High‐quality practice relies on established standards like pediatric endoscopy quality improvement network (PEnQuIN)^24^ and structured training with simulation, milestone assessment, e‐learning, and “train the trainer” programs.^25^ Challenges remain (i) limited child‐specific instruments, particularly for <10 kg, (ii) uneven access to advanced procedures due to scarce pediatric expertise, (iii) heterogeneous training, and (iv) unequal global access.

Internationally, collaborative initiatives led by European Society for Paediatric Gastroenterology, Hepatology, and Nutrition and North American Society for Pediatric Gastroenterology, Hepatology and Nutrition have emphasized the standardization of pediatric endoscopy quality indicators, competency‐based training, and harmonized reporting systems. These efforts aim to improve procedural safety, reproducibility of outcomes, and equitable access to advanced endoscopic technologies across centers.^23^, ^24^, ^25^

### 4.2 Goals and objectives

The WG's primary goals are to enhance procedural safety and effectiveness. Developing miniaturized and child‐specific equipment is essential. Integrating AI and computational imaging aims to improve diagnostic accuracy, support real‐time decision‐making, and facilitate remote training.^23^ Research should refine therapeutic procedures and validate training frameworks.

### 4.3 Research priorities

The SIGENP Endoscopy WG considers that future research priorities include validated quality‐reporting standards, improved devices for pediatric hemostasis, evaluation of anxiety, and long‐term safety of repeated procedures. Expanding therapeutic procedures (ERCP, EUS, and POEM) and incorporating AI into diagnostic and training workflows are key. International registries and equity of access, especially in low‐resource settings, must be strengthened, while environmental sustainability represents an emerging goal.

### 4.4 Plans

Planned strategies include expanding the national EndoPED registry, implementing simulators and e‐learning platforms for safer training, and collaborating with adult societies and industry on pediatric‐specific tools. Standardized audits based on PEnQuIN quality indicators and AI‐supported reporting will enhance reproducibility and quality assurance. Collaboration with international bodies will promote equitable, sustainable, child‐centered endoscopy.

## 5 HEPATOLOGY

Pediatric hepatology has progressed due to advances in genetics, imaging, biomarkers, and targeted therapies. Rare liver diseases—including progressive familial intrahepatic cholestasis, Alagille syndrome (AS), metabolic disorders, and immune‐mediated hepatopathies—require multidisciplinary and long‐term monitoring. Metabolically dysfunctional‐associated steatotic liver disease (MASLD) is increasingly prevalent. Despite progress, key challenges remain, including (i) inconsistent access to non‐invasive diagnostics, and (ii) limited therapeutic options for many conditions.

### 5.1 Current knowledge and gaps

Significant gaps persist in the understanding and management of both rare and common pediatric liver diseases. In genetic hepatopathies, next‐generation sequencing and universal metabolic screening have improved diagnostic yield, but clinical integration and interpretation of complex variants remain challenging. Gene therapy trials yet remain limited by vector toxicity, variable efficacy, and the absence of long‐term outcome data.^26^ In cholestatic disorders, ileal bile acid transporter (IBAT) inhibitors such as maralixibat and odevixibat have broadened therapeutic options, though their optimal timing, long‐term safety, and potential application to additional cholestatic diseases remain under investigation.^27^, ^28^, ^29^ In autoimmune liver disease (AIH) and primary sclerosing cholangitis continue to rely heavily on adult‐derived evidence; the lack of pediatric biomarkers and relapse predictors restricts precision medicine approaches.^30^, ^31^ In MASLD, alanine transaminase (ALT) alone is inadequate for diagnosis, and tools such as transient elastography and CAP still lack standardized pediatric thresholds.^32^ Polygenic risk variants including patatin‐like phospholipase domain–containing protein 3, transmembrane6 superfamily member 2 (TM6SF2), and membrane bound acylglicerophosphatydilinositol acyltransferasi (MBOAT7) are emerging as promising stratification tools.^32^

In vascular liver diseases, management of extrahepatic portal vein obstruction and congenital portosystemic shunts varies widely across centers, and procedures such as Meso‐Rex bypass, portal vein recanalization, and Transjugular Intraepathic portosystemic shunt (TIPS) require further multicenter validation.^33^ In liver transplantation, long‐term challenges include chronic immunosuppression toxicity, suboptimal adherence during adolescence, and insufficient understanding of the progression of graft fibrosis. In pediatric pancreatitis, key gaps include the absence of pediatric‐specific biomarkers, limited disease‐modifying therapies, and heterogeneity in pain and nutrition management strategies.

Globally, pediatric hepatology research is increasingly driven by multicenter collaborative networks focusing on rare diseases, translational genomics, and innovative therapeutics. International studies are expanding the application of multi‐omic approaches, gene‐based therapies, and biomarker‐driven disease stratification to improve diagnosis and long‐term outcomes in children with liver diseases.^26^, ^31^, ^32^

### 5.2 Goals and objectives

Pediatric hepatology research aims to advance integrated, precision‐driven care through multi‐omic approaches, longitudinal registries, and biomarker‐guided diagnostics. Novel therapies—including gene‐based, immunologic, and microbiome‐targeted strategies—are needed across liver diseases. Priorities include improving non‐invasive diagnostics, incorporating patient‐reported outcomes, and harmonizing care through guidelines and prospective studies.

### 5.3 Research priorities

Research should accelerate molecular diagnosis and optimize gene therapy strategies for inherited liver diseases,^26^ while also expanding long‐term evaluation of IBAT inhibitors across cholestatic phenotypes. Identifying immunologic predictors of relapse in autoimmune hepatopathies is essential to improve disease monitoring and guide therapy. In MASLD, standardizing diagnostic pathways and assessing emerging therapeutics—including thyroid hormone receptor‐β agonists and glucagon‐like peptide (GLP)‐1 analogs—is a priority.^34^ Further efforts should focus on establishing consensus‐driven management strategies for vascular liver diseases, strengthening post‐transplant immune monitoring and transition pathways, and developing biomarkers and therapeutic trials for pediatric pancreatitis.

### 5.4 Plans

Future hepatology research will depend on multicenter collaborative networks and national consortia capable of producing adequately powered pediatric studies. Innovative treatments—including gene editing, targeted biologics, metabolic modulators, and microbiome‐directed therapies—must be rigorously tested within pediatric regulatory and ethical frameworks. Engaging patients and families in study design will enhance adherence and translational impact across all research initiatives.

## 6 NUTRITIONAL DISORDERS AND INTESTINAL FAILURE

Recent years have seen significant advances in pediatric nutrition and intestinal failure management, with increasing attention to early‐life nutritional programming and strategies to address the growing burden of childhood obesity. In parallel, artificial nutrition has evolved through blended and real‐food formulas and GLP‐2 analogs such as teduglutide, supporting intestinal adaptation in children with intestinal failure.

The SIGENP Working Group has contributed substantially to the areas of EN for neurologically impaired children, intestinal failure, and severe nutritional disorders. Although multidisciplinary care has advanced, many children continue to require long‐term parenteral nutrition (PN), with risks including infections, growth impairment, and intestinal failure–associated liver disease. Evidence remains fragmented and often adult‐derived.

### 6.1 Current knowledge and gaps

Between 1990 and 2022, global nutrition shifted: underweight declined, while obesity surged.^35^ In the WHO European Region, ~60% of adults and one‐third of children are overweight/obese.^35^, ^36^ Obesity treatment includes lifestyle interventions, pharmacotherapy, and bariatric surgery.^37^ Pharmacotherapy is costly (€200–300/package), can cause serious GI events (~10%), and discontinuation often leads to weight regain. Bariatric surgery is most effective but carries a 20%–25% reoperation risk, anemia, micronutrient deficiencies, and bone loss.

Early‐life nutrition shapes long‐term health via the first 1000 days.^38^ Maternal diet, breastfeeding, and complementary feeding (CF) are crucial; early CF (<6 months) occurs in ~29% of infants in low‐middle income countries. Baby‐led‐weaning and Baby‐led introduction to solids (BLW/BLISS), October approaches may increase sodium/fat intake but not iron, and do not increase choking risk with training.^39^, ^40^ Early taste exposure promotes healthier dietary patterns; Mediterranean, Nordic, dietary approaches to stop hypertension, and Washoku diets link to better cardiometabolic outcomes.^41^ EN is essential but costly; PN can exceed hundreds of thousands of euros annually.^42^ Teduglutide (GLP‐2 analogue) reduces PN needs in short bowel syndrome; response predictors include residual bowel length, baseline nutrition, and early response.^46^

At the international level, pediatric nutrition research is increasingly oriented toward systems‐based approaches linking early‐life dietary exposures, microbiome development, and long‐term metabolic health. Large epidemiological studies and international nutrition networks are contributing to the understanding of global trends in childhood obesity and the long‐term impact of early nutritional programming.^35^, ^41^, ^42^

### 6.2 Goals and objectives

The Working Group prioritizes clarifying how early‐life nutrition affects obesity and GI disease. Key objectives include evaluating neonatal risk factors for allergic proctocolitis, assessing Mediterranean diet effects on obesity and GI outcomes, and refining best practices in CF. In artificial nutrition, systematic evaluation of blended and real‐food diets versus polymeric formulas is required to determine efficacy and safety. In intestinal failure, defining predictors of teduglutide responsiveness is essential.

### 6.3 Research priorities

Priorities include defining early‐life dietary determinants of obesity and GI disease, including microbiota pathways, and evaluating protective dietary patterns in large cohorts. Multicenter studies should assess enteral formulas, while registry and trial‐based research in short bowel syndrome should optimize predictors of PN weaning, growth, and teduglutide use.

### 6.4 Plans

Planned initiatives include foundation funding for neonatal nutrition research, European grant applications for Mediterranean diet and obesity risk studies, and industry partnerships for comparative EN studies. Research on short bowel syndrome and teduglutide responsiveness will continue through self‐funded multicenter registries on REDCap, ensuring integration of real‐world evidence with clinical practice.

## 7 FUNCTIONAL AND MOTILITY DISORDERS

GI motility and functional disorders, with the latter now defined as disorders of gut–brain interaction (DGBI) as classified by the Rome Criteria,^44^ are highly prevalent in pediatric populations. Advances in neurogastroenterology have improved understanding of the complex interplay between the enteric nervous system, microbiome, immune pathways, and psychosocial factors, encompassing conditions that range from DGBI^44^ to severe gut dysmotility.

### 7.1 Current knowledge and gaps

Esophageal reflux assessment has advanced with pH‐impedance monitoring, now the gold standard for gastroesophageal reflux disease (GERD) diagnosis.^45^ Pediatric non‐erosive reflux disease (NERD) is the most common GERD form, subdivided into “true” NERD, reflux hypersensitivity, and functional heartburn.^46^ Pediatric prevalence and management data are limited. Novel diagnostics from adult practice, such as functional lumen imaging probe (FLIP) and high‐resolution manometry (HRM), allow detailed esophageal function assessment.^47^ Pediatric normative HRM and FLIP values are still lacking. Achalasia therapies include pneumatic dilation, Heller myotomy, and POEM, which shows promising short‐term outcomes but limited long‐term data.

Gastric motility is evaluated by gastric emptying scintigraphy, with radiation‐free 13C‐breath tests emerging.^47^ Antro‐duodenal manometry is reserved for complex cases as suspected pediatric intestinal pseudo‐obstruction.^48^ Management includes early diagnosis, multidisciplinary care, nutrition, bacterial overgrowth treatment, and prokinetics. Functional GI disorders benefit from psychological therapies (cognitive behavioral therapy, CBT; gut‐directed hypnotherapy) and neuromodulation (percutaneous electrical nerve field stimulation, percutaneous electrical nerve field stimulation; gastric electrical stimulation), while dietary interventions like low‐FODMAP diets remain under investigation.

Anorectal and colonic function are assessed via anorectal manometry and colonic manometry.^49^ These techniques can help identify different underlying disorders and appropriate treatment approaches. Novel treatments for refractory constipation include pharmacological agents such as linaclotide and percutaneous posterior tibial nerve stimulation, which show efficacy also in pediatric patients.^50^

International research in pediatric neurogastroenterology increasingly integrates insights from microbiome science, neuroscience, and digital health technologies. Collaborative initiatives are promoting standardized diagnostic protocols and advancing the understanding of DGBI through multidisciplinary and multicenter studies.^44^, ^48^, ^49^

### 7.2 Goals and objectives

Future research must refine the understanding of phenotype‐specific pathophysiology and support individualized therapeutic strategies. In esophageal disorders, priorities include advancing diagnostic accuracy and tailoring therapy to non‐erosive phenotypes. In gastric and small bowel dysmotility, integrating multimodal assessment tools with novel pharmacologic and neuromodulatory approaches is essential. For colonic and anorectal disorders, validation of HRM combined with complementary imaging, including endoanal ultrasound and defecography, is needed.

### 7.3 Research priorities

Priorities include phenotype‐driven therapies for DGBI, multicenter registries for longitudinal data, and randomized trials on laxatives, neuromodulators, psychological therapies, and devices in pediatric GI disorders. Additional goals are generating pediatric‐specific diagnostic data, developing minimally invasive tools, and applying AI and big data to motility studies.

### 7.4 Plans

Progress requires interdisciplinary collaboration, minimally invasive diagnostics, and precision‐based therapies. Integration of big data and participation in multicenter networks will be key to translating insights into improved outcomes and personalized care.

## 8 ALLERGY AND IMMUNITY

Over recent years, the SIGENP Allergy Working Group has focused on immune‐ and non‐immune–mediated food‐related disorders. Early introduction of allergenic foods such as peanuts and eggs during infancy has emerged as an effective strategy to reduce food allergy risk, while the timing of gluten introduction does not appear to influence celiac disease onset.^51^ The rising prevalence of celiac disease and reliance on a strict gluten‐free diet continue to pose clinical and psychosocial challenges. Non‐allergic adverse reactions, including food refusal, pseudo‐dysphagia, and abdominal pain, may reflect individual variation in taste receptor and transient receptor potential vanilloid (TRPV) channel phenotypes.

### 8.1 Current knowledge and gaps

Evidence strongly supports early allergen introduction, particularly in high‐risk infants with eczema or pre‐existing allergies, where early exposure between four and 6 months reduces food allergy incidence.^52^ Celiac disease, meanwhile, continues to increase globally and is shaped by modifiable environmental factors—including infections, gluten load in the second year, mode of delivery, and antibiotic exposure—which influence epigenetic regulation of disease‐related genes.

The global prevalence of celiac autoimmunity is estimated at 1.4%, and screening initiatives are expanding detection. Updated diagnostic guidelines emphasize serological thresholds for anti‐transglutaminase immunoglobulin A and confirmatory anti‐endomysium positivity, reducing the need for biopsy, while human leukocyte antigen (HLA) typing is no longer required.^53^ Despite improved diagnostics, management still relies on lifelong gluten avoidance.

Investigational treatments aim to reduce gluten‐induced injury. Enzyme supplements such as latiglutenase and tissue transglutaminase 2 (TG2) inhibitors such as ZED‐1227 have shown encouraging early results in limiting mucosal damage.^54^ Stratification by HLA‐DQ2/DQ8 gene dosage may refine therapeutic response. Probiotics have demonstrated immunomodulatory effects, including reduced Th1‐driven inflammation and enhanced tolerogenic cytokines, though clinical benefit remains inconsistent. Immunotherapeutic vaccines such as Nexvax2 and CeliVax are under development to induce antigen‐specific tolerance. Organoid platforms—which recapitulate epithelial and immune interactions—have identified interleukin (IL)‐7 as a key gluten‐inducible mediator driving CD8^+^ T‐cell activation and epithelial damage in celiac disease.^55^ Monoclonal antibodies targeting IL‐15 and related pathways represent emerging options to counteract immune‐mediated epithelial injury.

International research efforts are also expanding through translational platforms integrating genomics, environmental exposure studies, and advanced experimental models. Organoid systems and immune profiling technologies are providing novel insights into epithelial–immune interactions and supporting the development of disease‐modifying therapies for celiac disease and other immune‐mediated GI disorders.^51^, ^52^, ^54^

### 8.2 Goals and objectives

A key objective is to deepen mechanistic understanding of celiac disease to guide prevention and therapy while optimizing current management. Organoid‐based in vitro models incorporating immune components offer a powerful platform to replicate patient‐specific responses. These systems enable detailed investigation of anti‐tTG antibody–mediated injury, assessment of immunosuppressive agents, TG2 inhibitors, and monoclonal antibodies, and preclinical evaluation of potential protective treatments. Such approaches support personalized medicine and may streamline development of disease‐modifying therapies.

### 8.3 Research priorities

Priorities include advancing organoid models to study patient‐specific epithelial–immune interactions for drug discovery, biomarker development, and preclinical validation. Research should also address environmental and epigenetic factors (e.g., early infections, antibiotics) and genetic determinants of food intolerance and functional GI disorders, including TAS2R38 and TRPV variants.

### 8.4 Plans

Future studies should leverage multicenter and translational platforms, including organoids, to investigate epithelial–immune mechanisms. Coordinated collaboration, longitudinal studies in at‐risk infants, and multicenter analyses integrating sensory and genetic data will be key to identifying modifiable risk factors and improving reproducibility.

## 9 CONCLUSIONS

Pediatric gastroenterology, hepatology, and nutrition are rapidly evolving fields, driven by increasing disease complexity, technological advances, and the growing need for pediatric‐specific evidence. Identifying research priorities is essential to guide collaborative efforts and accelerate the translation of emerging discoveries into clinical practice. The priorities outlined in this review highlight key knowledge gaps across multiple domains, including inflammatory diseases, hepatology, nutrition, endoscopy, and motility disorders. Although developed within the framework of a national scientific society, the priorities proposed here reflect challenges shared across pediatric gastroenterology worldwide and aim to stimulate international dialogue and collaborative research to improve outcomes for children with digestive disorders.

## CONFLICT OF INTERESTS STATEMENT

The authors declare no conflicts of interest.
